# Functional Polymorphisms in *PRODH* Are Associated with Risk and Protection for Schizophrenia and Fronto-Striatal Structure and Function

**DOI:** 10.1371/journal.pgen.1000252

**Published:** 2008-11-07

**Authors:** Lucas Kempf, Kristin K. Nicodemus, Bhaskar Kolachana, Radhakrishna Vakkalanka, Beth A. Verchinski, Michael F. Egan, Richard E. Straub, Venkata A. Mattay, Joseph H. Callicott, Daniel R. Weinberger, Andreas Meyer-Lindenberg

**Affiliations:** 1 Unit of Systems Neuroscience in Psychiatry, National Institute of Mental Health, Department of Health and Human Services, National Institutes of Health, Bethesda, Maryland, United States of America; 2 Genes, Cognition, and Psychosis Program, Clinical Brain Disorders Branch, National Institute of Mental Health, Department of Health and Human Services, National Institutes of Health, Bethesda, Maryland, United States of America; 3 Neuroimaging Core Facility, National Institute of Mental Health, Department of Health and Human Services, National Institutes of Health, Bethesda, Maryland, United States of America; 4 Central Institute of Mental Health, Mannheim, Germany; Johns Hopkins University School of Medicine, United States of America

## Abstract

*PRODH*, encoding proline oxidase (POX), has been associated with schizophrenia through linkage, association, and the 22q11 deletion syndrome (Velo-Cardio-Facial syndrome). Here, we show in a family-based sample that functional polymorphisms in *PRODH* are associated with schizophrenia, with protective and risk alleles having opposite effects on POX activity. Using a multimodal imaging genetics approach, we demonstrate that haplotypes constructed from these risk and protective functional polymorphisms have dissociable correlations with structure, function, and connectivity of striatum and prefrontal cortex, impacting critical circuitry implicated in the pathophysiology of schizophrenia. Specifically, the schizophrenia risk haplotype was associated with decreased striatal volume and increased striatal-frontal functional connectivity, while the protective haplotype was associated with decreased striatal-frontal functional connectivity. Our findings suggest a role for functional genetic variation in POX on neostriatal-frontal circuits mediating risk and protection for schizophrenia.

## Author Summary

Schizophrenia is a major mental illness affecting 1% of the population. It is known that genetics plays a role in the disease susceptibility, and it is thought that the illness is a complex disorder involving multiple genes. We show that the schizophrenia susceptibility gene, *PRODH*, conveys its risk through a variation that increases its enzyme activity. We further show that protection is associated with variations that decrease enzyme activity and these protective variations are enriched in their unaffected siblings. We then used brain imaging of structure and memory function to dissect the risk and protective haplotypes differential effects, and found that the schizophrenia risk haplotype was associated with decreased striatal gray matter volume and increased subcortical to frontal lobe functional connectivity, while the schizophrenia protective haplotype was associated with trend-level increase of frontal lobe volume and decreased subcortical to frontal lobe connectivity. These findings indicate a new target for treating schizophrenia and characterize associated structural and functional deficits.

## Introduction

Schizophrenia is a debilitating illness affecting about 1% of the population with a $62.7 billion estimated economic burden in the United States alone [1]. Twin studies and adoption studies have shown that disease risk is largely genetic, though characterizing the neural mechanisms underlying the disorder and mediating this genetic risk is a challenging process. Certainly, the genetic architecture of schizophrenia is complex and involves multiple genes. A candidate gene for schizophrenia that has received extensive investigation, with conflicting results, is *PRODH*, encoding proline oxidase (POX). This enzyme, among other functions, is rate limiting in the conversion of proline to glutamate in mitochondria. *PRODH* along with its pseudogene are located at 22q11 is implicated in schizophrenia through a hemideletion syndrome at this locale, 22q11 deletion syndrome (22q11DS, Velo-Cardio-Facial syndrome, VCFS: OMIM #192430), which may be the strongest known risk factor for schizophrenia besides having a monozygotic twin with the disorder [2]–[6]. Furthermore, a linkage peak at 22q11 has also been identified in a meta-analysis of families with multiple affect patients with schizophrenia [7]. Several recent studies have found genetic associations of schizophrenia with tag and functional single nucleotide polymorphisms (SNPs) in *PRODH* [8]–[11], although this association has not been observed in all samples [12]–[18]. In addition to these genetic data, hyperprolinemia has been noted in populations of psychotic patients [19]–[26] and has been associated with neurological problems in 22q11 syndrome [24].

Recently, Bender et al [27] in a sample of European descent characterized several common functional genetic polymorphisms in POX that influence a wide range of enzymatic activity from reductions to below 30% of the reference sequence to increasing activity to 120% of the reference sequence. These findings offer the opportunity to study the impact of functionally characterized variation in POX on risk for schizophrenia.

Statistical genetic association studies can provide a link between genes and complex polygenetic constructs like mental illness, but this approach does not illuminate the possible underlying pathophysiology impacted or the mechanisms of association. Here we used a staged investigation ranging from clinical genetic association to studies of the associated pathophysiology through a multimodal imaging approach to examine the impact of variation in *PRODH* on risk for schizophrenia and function and structure in human brain of neural circuitries implicated in the pathophysiology of schizophrenia. First, we identified variants in *PRODH* related to risk for schizophrenia in a family-based sample. Then, we used imaging genetic techniques to investigate genetically correlated physiology by identifying neural systems on which these variations impact in normal subjects who carry risk associated alleles or haplotypes [28]. Investigating susceptibility genes in the normal population allows for the investigation of complex trait susceptibility alleles without confounding factors of disease, drugs of abuse or treatment effects. In this study, we used Voxel Based Morphometry (VBM) for whole brain quantitative structural analysis, and a robust working memory task (NBACK) previously shown to be sensitive to genetic variation associated with schizophrenia susceptibility [29],[30].

## Results

### Genetics

We found that the functional variants in *PRODH* were associated with risk for schizophrenia. (Table 1) Single marker analysis revealed that the minor allele for rs450046 was most strongly positively associated with schizophrenia in families. This allele has previously been associated with increased POX enzyme activity [27]. Conversely, the minor alleles of rs4819756 and rs2870983, linked to decreased POX activity [27], were significantly negatively associated with schizophrenia. Three-SNP sliding window haplotype analysis confirmed these separate individual SNP associations by showing change from positive associations to negative associations for haplotypes as they differentially included the 2 functional SNPs, rs2870983 and rs450046 separated by a tag SNP rs385440, in the 3′ prime end of the gene. The CAC haplotype at these three SNPs was positively associated and TGT was negatively associated with schizophrenia, as seen clearly in Table 1. Haplotype analysis comprised of the 3 functional SNPs (hereafter referred to as the “functional risk or protective haplotypes”) showed a significant positive disease association with the reference alleles for rs4819756 and rs2870983 and rs450046 minor alleles (risk haplotype: GCC) and negative association with haplotype of the minor allele for rs4819756 and the major alleles of rs2870983 and rs450046 (protective haplotype: ACT). In a post hoc analysis to test if the SNPs were truly protective, we tested whether excess transmission to unaffected siblings of schizophrenic patients was observed with protective alleles. We found a trend for the major T allele of rs450046 towards over transmission (z score = 2.142, p = 0.077). The results of the case control analysis, comparing unaffected siblings with unrelated controls, revealed a genotypic effect for the protective SNP rs4819756, with a heterozygote odds ratio of 1.8 (95% C.I.1.17–2.90, OR p value = 0.009) and a minor allele homozygote odds ratio of 2.3 (95% C.I.1.26–4.27, OR p value = 0.007). There was a significant association with the protective haplotype (ACT) and a negative association with the reference haplotype (GCT) (Table 2).

**Table 1:** Single SNP and Haplotype Clinical Association Results.

SNP identifier	Alleles	MAF*	Location/Enzyme activity	FBAT Z-score **	Haplotype				Functional Haplotype	
rs9604911***	G/T	0.440	5′ region							
rs8137125***	T/G	0.208	intron 2							
rs4819757***	T/C	0.166	intron 2							
rs17743056 ***	C/T	0.063	intron 3							
rs367766***	T/C	0.115	intron 3	1.798				**C**		
				P = 0.091						
rs2016108***	T/C	0.402	intron 3					**T**		
rs4819756	G/A	0.370	Exon 5 R185W	2.153				**A**	**G**	**A**
			30–70%	P = 0.043						
rs5746640***	G/A	0.438	intron 5							
rs16983466***	G/A	0.159	Exon 12 D426							
rs2870983	C/T	0.100	Exon 12 A472T	2.305		**C**	**T**		**C**	**C**
			<30%	P = 0.033						
rs385440***	G/A	0.161	intron 13	−2.110	**A**	**A**	**G**			
				P = 0.024						
rs450046	T/C	0.120	Exon 14 Q521R	−2.409	**C**	**C**	**T**		**C**	**T**
			120%	P = 0.026						
rs372055***	A/G	0.350	Exon 15 L581		**G**					
Association					Positive	Positive	Negative	Negative	Positive	Negative
Haplotype p value					0.007	0.03	0.019	0.01	0.007	0.022
Haplotypes Global p value					0.053	0.027	0.019	0.010	0.011	0.011

*MAF: minor allele frequency from dbSNP build 126 European population and **FBAT Z score for major allele with uncorrected P.

***: Hapmap Tag SNP.

**Table 2:** Sibcentric Results.

SNP identifier	FBAT*	P	Sib Control OR [95% CI] heterozygote	Homozygote	Functional Haplotype	
rs4819756	1.701	0.087	1.8 [1.16–2.90]	2.3 [1.26–4.27]	G	A
			P = 0.009	P = 0.007		
rs2870983					C	C
rs450046	2.142	0.077			T	T
Z-score					−2.96	2.77
Haplotype p value					0.003	0.005
Global p value					0.062	0.062

*: Z-score for major allele.

### Structural Imaging

The functional risk-associated haplotype (GCC) was associated with reduced regional gray matter volume in the neostriatum in the normal control sample (21, 22, −6, z = 4.92, P<0.05 whole brain corrected false discovery rate (FDR) (Figure 2 A and B). In a post hoc exploratory analysis, the protective haplotype (ACT) revealed a non significant trend towards gray matter volume increase in the frontal lobes (−21, 53, 8, z = 3.97, P<0.001 uncorrected) (Figure 2 A and B) and occipital lobes (21,−85, 17, z = 4.26, P<0.001 uncorrected) (Table S1)[Fig pgen-1000252-g001].

**Figure 1 pgen-1000252-g001:**
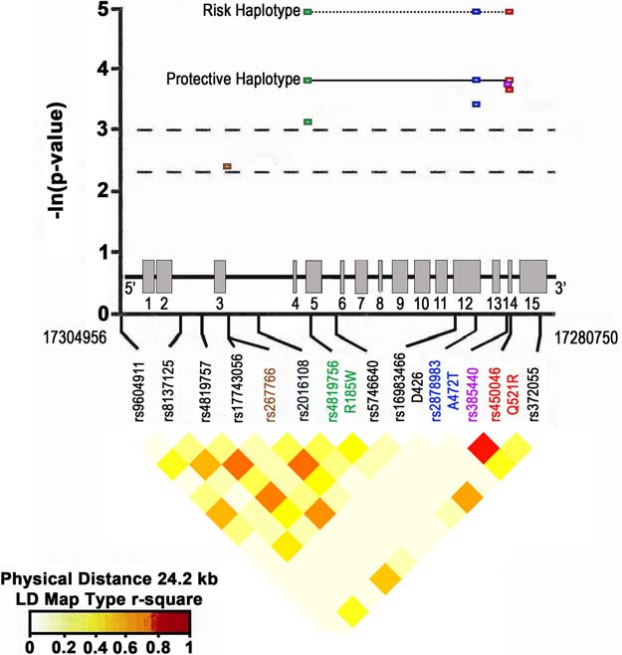
Genetic Association results. Above) −ln(p) clinical results for significant SNPs and functional haplotypes. Non-synonymous functional coding SNPs are labeled with coding changes. Exon structure is for most common transcript. Below) r^2^ heat map for CBDB normal controls of European descent as visualized in snp.plotter demonstrates the relatively low level of linkage disequilibrium across this region.

**Figure 2:**
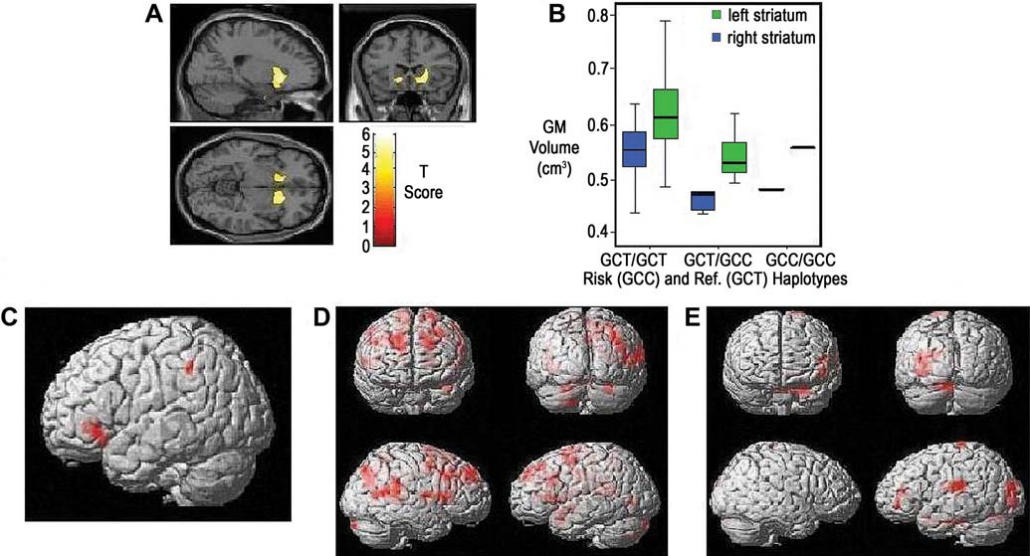
Risk and Reference haplotypes: multimodal imaging results (see supplemental tables for results surviving multiple comparison correction). A) Decreases in grey matter volume for risk haplotype carrier in relationship to the reference haplotype, at p<0.001 uncorrected threshold. B) Extracted striatal cluster grey matter values plotted by haplotype. C) Areas of relatively decreased BOLD signal for risk haplotype vs. reference effect on the working memory network in normal subjects in the working memory network visualized at uncorrected p<0.001 (VLPFC) k = 13 Z = 3.39, p<0.045 small volume corrected for VLPFC (45, −45, 42) k = 31 Z = 3.81, p<0.043 small volume corrected BA 40. Df) Area of relatively increased and E) decreased striatal connectivity for the risk haplotype at uncorrected p<0.05. Statistical mappings are overlaid on a single subject T1 images. Statistical results after multiple comparison correction shown in Tables S1, S2 and S3.

### Brain Function

**Figure 3:**
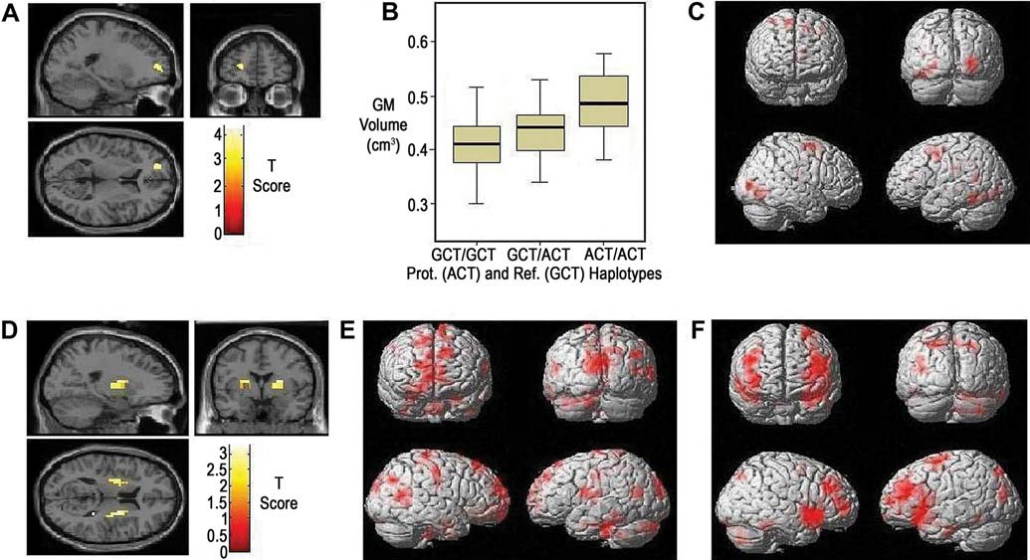
Protective and reference haplotypes: multimodal imaging results (see supplemental tables for results surviving multiple comparison correction). A) Increases in grey matter volume for protective haplotype carriers in relationship to reference haplotype at p<0.001 uncorrected threshold. B) Extracted values of cluster grey matter for left frontal lobe at threshold of p<0.001 as a function of protective haplotype. C) Relatively decreased BOLD signal of early sensory processing stream for protective haplotype carriers on the working memory network in normal subjects at FDR<0.047 full brain corrected. D) Decreased BOLD signal for protective haplotype carriers in relationship to reference haplotype, at p<0.05 uncorrected threshold within Caudate and Putamen as defined by the Wake Forest University brain atlas. Areas of relatively E) increased and F) decreased connectivity for protective haplotype effect at uncorrected threshold of p<0.05. Statistical mappings are overlaid on a single subject T1 images. Statistical results after multiple comparison correction shown in Tables S1, S2 and S3.

To further characterize these findings functionally, we analyzed the effect of the risk haplotype during working memory in the normal sample. Network activation patterns differed, with reduced blood oxygenation level-dependent (BOLD) signal in ventral lateral prefrontal cortex (VLPFC, z = 3.18, p<0.05 corrected) and parietal lobes (Brodmann area (BA) 40, z = 3.52, p<0.05 corrected) (Figure 2C) relative to the reference haplotype. (Table S2) Functional connectivity for the bilateral striatal seed regions showed trends for increased dorsal lateral prefrontal-striatal functional connectivity (Figure 2D) for the risk haplotype carriers. (Table S3) In contrast, the protective haplotype carriers had decreased BOLD in bilateral striatum compared to reference (−22, 0, 12, z = 3.23, p = 0.05 corrected) (Figure 3D), decreased recruitment of areas of early visual processing (BA 18, fusiform gyrus, BA6, BA19, z = 3.92, p<0.05 whole brain corrected) (Figure 3C) (Table S4) and a decreased striatal-frontal functional connectivity, z = 3.75, p<0.037 corrected for region of interest (ROI) (Figure 3E and F) (Table S3). Performance was not significantly different between haplotype groups (Table S5). Post hoc analysis including performance as a nuisance covariate had similar activation patterns.

### Control Experiment

In order to assess whether the impact on POX function is a likely proximal cause of the systems-level findings reported above, we performed the same set of imaging analyses with haplotypes constructed from the 3 SNPs with no observed clinical association or known function. These did not yield any significant genetic effects for all phenotypes studied above.

## Discussion

Using a translational approach, we show that functional polymorphisms in *PRODH*, encoding POX, are linked to risk for schizophrenia and associated with alterations in prefrontal-striatal brain circuits involved in working memory and cognitive gating. We found a positive disease association with the SNP previously shown [27] to increase enzyme activity and a negative association between 2 polymorphisms that decreased POX activity. One of the strengths of the present study is that it assesses direct association of multiple functional variants with disease status, instead of relying on linkage disequilibrium (LD) between non-functional variants and unobserved unknown variants; this is especially important given that previous association studies did not test multiple functional SNPs. Another advantage of this approach particularly in this gene is that tag SNP mapping in this gene is complicated by the presence of a pseudogene in the same chromosomal region leading to low population SNP to SNP r^2^ [31]. Additionally, in the functional assays of POX, it has been observed that the enzymatic activity resembles the lowest activity polymorphism when there is more than one in the haplotype [27]; therefore, without testing functional haplotypes the association may not be observed. Despite the compelling finding that directionality of disease association corresponded to the effects on enzyme activity, our data do not exclude effects from variants in long range linkage disequilibrium. However, the diminished LD structure of this area of the genome and the results of our three SNP sliding window analysis make this unlikely, as do the results from our control experiment showing that nonfunctional variants had no significant effect on brain phenotypes. We also tested post hoc the hypothesis that putative protected alleles should be enriched in an unaffected sibling population. Indeed, we found that unaffected sibling status is positively associated with the POX variants that decrease the enzyme's activity.

There are several reasons why this observation was difficult to appreciate in the previous literature. In the original finding, Lui et al [10] found a three SNP haplotype including one of the functional SNPs rs450046 positively associated with schizophrenia. Williams et al in two studies [15],[16] looked first at only the 3′ end of the gene with rs383964, rs372055, and rs450046 in 677/679 case control sample from the UK and the Irish Republic and 55 trios from Bulgaria. While they tested directly the increased enzymatic activity variant, they may not have observed positive results due to not testing the other decreasing activity SNPs and/or the difference in the haplotype structure in case/control sample. Their second study [16] used a more extensive map of SNPs but used a 368/368 case/control pooling method that would make inferring functional haplotypes impossible. Fan et al [17] typed rs372055 in a small Chinese family trio sample and observed no association in their sample. Li et al [8] found rs385440 and two-marker haplotypes consisting of rs385440 and rs450046 (G-G) and rs372055 and rs385440 (T-A) associated with schizophrenia in a 528 trio and sibling pair sample. Abu Jamra and colleagues [18] found no association with the three putatively non functional SNPs, rs16983466, rs372055, and rs383964, individually or in haplotypes in a German sample. Glaser et al [12] typed 4 SNPs (rs450046, rs383964, rs372055 and rs385440) in a sample of 488 Bulgarian trios. They determined that only 2 SNPs could be used by calculating r^2^ in a subsample and therefore typed only rs383964 and rs372055 and were not able to find an association in their full sample. This fact may reflect a difference in linkage disequilibrium in the greater sample or confounding by the other functional variants. Recently in a genome wide case control association study, Sullivan and colleagues using a large schizophrenia treatment study sample found a positive association for one of the three SNPs in *PRODH* at p = 0.023, but in the setting of a genome wide association study were unable to reach genome wide significance [11].

Our imaging findings provide further genetic evidence for fronto-striatal loop dysfunction as part of the pathophysiology of schizophrenia. This neural circuit has been previously defined in the animal [32] and human [33] literature as comprised of parallel, but interacting cortical-subcortical loops highly relevant for sensorimotor, emotional and cognitive performance. While the majority of our previous imaging genetic studies have focused on cortical working memory function “inefficiency” signal for increased activation of the dorsal lateral prefrontal cortex (DLPFC) with the recruitment of the VLPFC when performance is maintained, we are beginning to observe striatal to frontal “bottom up” processes also implicated in the pathophysiological processes. We previously have shown that polymorphisms in the *PPP1R1B* and in *AKT1* linked to increased striatal frontal connectivity and decreased striatal volume were associated with genetic risk for schizophrenia [34]–[36]. Here, we again observe decreased striatal gray matter volumes in carriers of the risk haplotype in concert with increased functional connectivity with DLPFC. Interestingly, risk haplotype carriers with evidence for altered striatal structure and connectivity (decreased striatal activations coupled with an increase of striatal to dorsal lateral prefrontal functional connectivity) showed alterations in a working memory network that suggest a decreased engagement of the VLPFC and parietal cortex. Paralleling these results, Tan and coworkers, using the same functional paradigm as here in schizophrenia, found evidence for shifts to cortically hierarchical lower level working memory processes (the VLPFC and parietal cortex) in the setting of DLPFC dysfunction and related to genetic risk [37]. Tan et al (2007) further showed that an epistatic genetic interaction of risk variants for schizophrenia involving the cortical glutamate and dopamine systems revealed similar regions of activation as seen here in healthy controls carrying *PRODH* risk variants but in the opposite direction with the two risk variants in *COMT* and *GRM3* showed increased engagement of the parietal cortex and VLPFC while *PRODH* risk haplotype showed an increase in striatal activity and decrease in activation of the parietal lobe and VLPFC [38]. This observed result may therefore be related to the interaction of the dopamine and glutamatergic systems through the POX enzyme but through the frontal-striatal loops. Of note, is that *PRODH* haplotypes groups had no significant performance differences and post hoc analysis including performance as a nuisance covariate did not change the activation patterns. Taken together, these convergent findings strongly support the concept that altered prefrontal-striatal processing is a genetic risk mechanism for schizophrenia [28],[35].

In contrast to the risk haplotype, the protective haplotype had opposing correlations on striatal functional connectivity (bottom-up processing) in the performing the Nback task. In addition, the protective haplotype was associated with trend-level increases in frontal gray matter, a finding that is of interest since prefrontal cortex is one of the areas in which reduced gray matter volume is most consistently found in schizophrenia [39]–[43]. This finding, however, must be viewed with caution as it did not meet stringent statistical thresholds. If independently confirmed in future studies, this genetic-structural association could suggest that allele-associated increases in prefrontal grey matter volume or integrity could contribute to a protective effect through this intermediate phenotype. Though not hypothesized, we speculate that our observation of increased efficiency of early visual processing areas seen for the protective haplotype could correspond to long range prefrontal cortex control on the early visual processing stream previously shown to be a “top-down” mechanism relevant for attentional selection [44] and therefore be related to more efficient prefrontal control of attentional resources.

At this time, while our experiments and control experiments suggest that it is the impact on POX function that is the proximal event linking genetic variation in *PRODH* to risk for schizophrenia, brain structure and function, the biochemical and cellular events mediating these observations are unknown. However, several mechanisms can be speculated about as a point of departure for further studies. First, L-Proline can directly modulate glutamatergic transmission in the brain and selective expression of a brain specific high-affinity proline transporter (*SLC6A7*) occurs in a subset of glutamatergic synapses [45]. Dysfunction in glutamatergic neurotransmission has been widely implicated in the pathophysiology of schizophrenia [46]. In a mouse model of type 1 hyperprolinemia, disruption of glutamatergic transmission was observed together with altered cortical dopamine transmission and increased catechol-o-methyltransferase gene expression [47]. The protective SNPs decrease POX enzyme activity and may increase levels of the precursor and possibly decrease glutamate synthesis in sensitive neuronal populations. Secondly, POX plays a role in a redox shuttle in the mitochondria, while also interacting with the intrinsic and extrinsic pathway of apoptosis [48],[49]. Mitochondrial dysfunction has been implicated in schizophrenia through expression data [50],[51] and abnormalities with redox reactions have been associated with schizophrenia [52]. Finally, Proline is also a recognized osmolite and it has been shown recently that increased intracellular proline content modulates stability and aggregation of polyglutamate proteins [53]. While the patho-etiological evidence for a polyglutamate process in schizophrenia is limited [54], several intracellular structural proteins have previously been associated with this disorder [55]. Molecular mechanisms unknown at this time may also contribute to this association, for example, serine racemase is not simply an enzyme to produce D-serine. D-amino acid oxidase does not simply degrade D-serine, but also degrade other D-amino acids.

Our results appear inconsistent with earlier associations of overt hyperprolinemia and psychosis [20],[21],[25]. We believe this discrepancy may be more apparent than real. Notably, blood proline levels have a wide normal distribution [25]. We found that common variants that would presumably translate into relative increases of proline levels in the normal range were protective, while risk was associated with the common variants that decreased levels in the normal range; therefore, our results raise the possibility that increased risk of psychosis in the context of clinical hyperprolinemia [20],[21],[25] operate through different molecular mechanisms; for example, proline's ability to directly activate glutamatergic NMDA receptors at extremely high levels [25] or hypoglutamatergic neuronal state due to the lack of the metabolic precursor, Δ′-pyrroline-5-carboxylic acid or other cellular role of POX. Many other roles of POX in the cell are still being discovered [56]–[58] and will been illuminating for discovering the necessary molecular mechanism that POX increases susceptibility for schizophrenia.

In conclusion, our data provide evidence that *PRODH* genetic variations that modulate the enzymatic activity of the POX enzyme contribute to the risk of schizophrenia through an impact on fronto-striatal processing. Our results may offer a novel target for the development of future therapeutic interventions.

## Materials and Methods

### Clinical Sample

For the genetics studies, 303 probands with schizophrenia spectrum disorders, their unaffected siblings, their parents and 370 controls were studied as part of the Clinical Brain Disorders Branch Sibling Study (Protocol 95-M0150, DRW PI) at the National Institute of Mental Health. All subjects gave written informed consent before participation in accordance to the Internal Review Board of the NIMH. Only subjects of European ancestry were used in the present study to minimize effects of population stratification. An independent sample of psychiatrically and neurologically screened healthy individuals was used for the neuroimaging control data sets (Table S5).

### SNP Selection

4 common functional nonsynonymous SNPs and one synonymous SNP, previously associated with schizophrenia, were studied (Table 1). For additional coverage of the gene, fourteen additional tag SNPs were chosen from the Hapmap project CEU data for NCBI build 36 (dbSNP build 126) [59]. For selection of the tagging SNP set, we used a 2–3 SNP aggressive tagging algorithm [60] as implemented in **Haploview** with minor allele frequency >0.05 and r^2^>0.80 [61]. One tag SNP failed on design of the genotype assay, 3 tag SNPs including one functional SNP failed due to poor amplification and one functional SNP and 3 tag SNPs failed due to non specificity because of the pseudo-*PRODH* gene detected by deviance from Hardy-Weinberg equilibrium >0.05.

### Genotyping

We used standard methods to extract DNA from lymphoblastoid cell lines using the Puregene DNA purification kit (Gentra Systems, Minneapolis, MN). *PRODH* genotyping was performed using the Taqman 5′-exonuclease allelic discrimination assay [62] obtained from Applied Biosystems (Foster City, CA) with primers and probes sets from Assays by Design. LD mapping and association test result plotting was performed using the R package snp.plotter [63].

### Imaging Haplotype Construction

We used a Bayesian method for haplotype construction (Phase v2.1)[64],[65] using the full Caucasian normal control sample (N = 368) for the remaining 13 SNPs using 5000 iterations, one thinning interval and a burn-in of 1000. We then recoded the inferred haplotypes in relationship to the 3 functional SNPs; rs450046, rs2870983 and rs4819756. 259 individuals were considered for imaging analysis if the functional haplotype were inferred at a greater than 90 percent probability. Only the reference, risk and one protective haplotype existed at appreciable population frequency rates >5% for haplotype imaging analysis. 17 of the 132 individuals with Nback data and 20 of the 124 individuals with VBM data were excluded due to missing data of a functional SNP which resulted in ambiguous haplotype assignment.

As a control experiment, to ascertain whether the impact on POX function is a likely contributor to our findings, we performed this procedure for three SNPs that had no clinical association, no known function (in particular, no impact on POX activity) and r^2^<0.2 with any known functional SNP, rs9604911, rs17743056, and rs5746640 and analyzed these haplotypes exactly as stated above. 64 individuals were used in the VBM analysis and 55 were used in the Nback analysis.

### Genetic Association

Family-based association testing was done via FBAT for single SNPs, 3-SNP sliding window haplotypes and haplotypes comprised of the three functional SNPs. All p-values were obtained via permutation testing using 1,000 replicates and are not adjusted for multiple testing.

If the protective haplotype decreases risk for schizophrenia, it should be overtransmitted from the parents to unaffected siblings of schizophrenia. Therefore, we additionally performed a post hoc “sibcentric” analysis, by considering the transmissions to the unaffected siblings to be the “case” and non-transmission of parental alleles to be “pseudocontrols” of interest using FBAT (n = 235 families) and also examined them in a logistic regression using only one unaffected sibling per family single functional SNP genotypic case/control (n = 167/350) method in STATA and with a 3 SNP functional haplotype analysis using the R package haplo.stats [66].

### Structural MRI

For structural analysis, scans for 92 individuals passed quality control and also had usable genotypes. Of those, 39 subjects were included in the risk compared to reference haplotype analysis and 85 subjects were included in the protective compared to reference haplotype analysis, Table S5. Structural scans were performed on a 1.5 T GE scanner (General Electric, Milwaukee, WI) using a T1-weighted SPGR sequence (repetition time, 24 msec; echo time, 5 msec, 256×256; field of view, 24×24 cm; flip angle 45°), with 124 sagittal slices at a thickness of 1.5 mm and an in-plane resolution of 0.94×0.94 mm. Optimized VBM [67],[68] was performed using custom templates in SPM2 http://www.fil.ion.ucl.ac.uk/spm/; (Wellcome Department of Imaging Neuroscience, London, UK) as previously described [69]. Modulated and segmented grey matter images were smoothed with a 12-mm Gaussian kernel prior to statistical analysis.

### Functional Neuroimaging

The Nback task has been shown to reliably activate the working memory network in normal subjects, shows an abnormal pattern in schizophrenia patients and their healthy relatives and is sensitive to genetic variation [30],[70]. Briefly, we used a block design version of the Nback task where 100% of stimuli were both target and probes. A 0-back control task block where the subject simply responded with the current digit presented (1–4 in a diamond shaped box) was alternating with the 2-back block in which the subject serially responded with numbers presented 2 previous (“n” = 2). Data were available for 108 subjects. 48 subjects were used in the risk compared to reference haplotype analysis and 103 subjects were used in the protective compared to reference haplotype analysis (Table S5). BOLD fMRI was performed on a GE Signa 3-T scanner using gradient echo EPI (24 axial slices, 6 mm thickness, 1 mm gap, TR/TE = 2000/28 ms, FOV = 24 cm, matrix = 64×64). Images were processed on the first level using SPM99 with a 2>0 back contrast.

### Functional Connectivity Analysis

Based on structural findings of the risk haplotype, which identified striatum, prefrontal connectivity with striatum was characterized using BOLD response maps with the Nback task by computing functional connectivity, as described previously [35]. Briefly, seed ROI for functional connectivity were placed within the combined bilateral caudate, head and body, and putamen as defined in the Wake Forest University brain atlas (WFU) (www.fmri.wfubmc.edu) [71], and median BOLD time course in these regions were then correlated across all voxels, yielding a map of correlation coefficients in every voxel with the seed region activity. These functional correlation maps were then analyzed in a random-effects model in SPM for haplotype effects as below.

### Second Level Analysis

Second level linear regression analyses as implemented using the general linear model in SPM2 of structural data and SPM99 for functional data [72]. Imputed *PRODH* haplotypes were covariates of interest entered into the model with the following nuisance covariates: for VBM: gender, total gray matter volume, age, and second-order polynomial age expansions and for Nback: age and gender. For all analyses, a stringent threshold of p<0.05, corrected for multiple comparisons using false discovery rate, was used, either for whole brain or small volume corrected for a priori hypothesized ROI. ROI analysis for the Nback was based on known regions involved in functional compensatory mechanisms during Nback working memory as previously described in Tan et al. [37] and regions engaged in gene×gene interaction of glutamate signaling and dopamine systems [73]. In addition, the bilateral striatum ROI constructed as above was used to examine haplotype dependent BOLD signal. Based on our previous genetic findings [35] for striatal frontal connectivity, analysis was restricted to ROI of dorsal lateral prefrontal cortex (DLPFC BA 45, 46, 9). We have recently shown that this methodology affords excellent protection against false positives in imaging genetics [74].

## Supporting Information

Table S1 Morphometry results.
(0.03 MB DOC)

Table S2 Nback working memory reference>risk haplotypes.
(0.03 MB DOC)

Table S3 Striatal functional connectivity.
(0.03 MB DOC)

Table S4 Nback working memory protective<reference haplotypes.
(0.05 MB DOC)

Table S5 Demographics.
(0.04 MB DOC)
