# Supplementary material for: Functional Polymorphisms in PRODH Are Associated with Risk and Protection for Schizophrenia and Fronto-Striatal Structure and Function
Source: PLoS Genet. 2008 Nov 7;4(11):e1000252. doi: 10.1371/journal.pgen.1000252 (PMC2573019; doi:10.1371/journal.pgen.1000252)
Supplement: Table S1 — Morphometry results. (0.03 MB DOC) [file pgen.1000252.s001.doc]

**Table S1**

Morphometry

|  | Voxel-level FDR | T | Z | P  uncorrected | X,Y,Z mm | K | Region |
| --- | --- | --- | --- | --- | --- | --- | --- |
| *reference>risk* | 0.041 * | 5.98 | 4.91 | <0.001 | 21,22,-6 | 978 | Putamen |
| *protective>reference*  ROI superior  Frontal lobe | 0.064 + | 4.34 | 4.12 | <0.001 | -21,53,8 | 6004 | BA 10 |
