# Supplementary material for: Functional Polymorphisms in PRODH Are Associated with Risk and Protection for Schizophrenia and Fronto-Striatal Structure and Function
Source: PLoS Genet. 2008 Nov 7;4(11):e1000252. doi: 10.1371/journal.pgen.1000252 (PMC2573019; doi:10.1371/journal.pgen.1000252)
Supplement: Table S2 — Nback working memory reference>risk haplotypes. (0.03 MB DOC) [file pgen.1000252.s002.doc]

**Table S2**

Nback working memory reference>risk haplotypes

|  | Voxel-level FDR | T | Z | P  uncorrected | X,Y,Z  mm | K | Region |
| --- | --- | --- | --- | --- | --- | --- | --- |
| ROI BA 44,45,47 | 0.045 + | 3.39 | 3.18 | 0.001 | 45, 22, -12 | 1 | VLPFC |
|  | 0.045 + | 3.37 | 3.16 | 0.001 | 30, 38, -6 | 1 |  |
|  | 0.045 + | 3.10 | 2.93 | 0.002 | 52, 19, 12 | 13 |  |
|  | 0.045 + | 3.10 | 2.93 | 0.002 | 30, 26, -18 | 11 |  |
| ROI BA 40 | 0.043 + | 3.81 | 3.52 | <0.001 | 45, -45, 42 | 31 | BA 40  Region of  Conjunction  for *GRM3*  and *COMT* |
