# Supplementary material for: Functional Polymorphisms in PRODH Are Associated with Risk and Protection for Schizophrenia and Fronto-Striatal Structure and Function
Source: PLoS Genet. 2008 Nov 7;4(11):e1000252. doi: 10.1371/journal.pgen.1000252 (PMC2573019; doi:10.1371/journal.pgen.1000252)
Supplement: Table S3 — Striatal functional connectivity. (0.03 MB DOC) [file pgen.1000252.s003.doc]

**Table S3**

Striatal functional connectivity

|  | Voxel-level FDR | T | Z | P  uncorrected | X,Y,Z mm | K | Region |
| --- | --- | --- | --- | --- | --- | --- | --- |
| *protective<reference*  ROI DLPFC BA  45, 46, 9 | 0.037 + | 3.91 | 3.75 | <0.001 | -45, 41, 24 | 36 |  |
| *reference<risk*  ROI DLPFC BA  45, 46, 9 | .268 + | 2.88 | 2.88 | 0.003 | 26, 49, 42 | 16 | Multiple diffuse non contiguous voxels |
