# Supplementary material for: Functional Polymorphisms in PRODH Are Associated with Risk and Protection for Schizophrenia and Fronto-Striatal Structure and Function
Source: PLoS Genet. 2008 Nov 7;4(11):e1000252. doi: 10.1371/journal.pgen.1000252 (PMC2573019; doi:10.1371/journal.pgen.1000252)
Supplement: Table S4 — Nback working memory protective<reference haplotypes. (0.05 MB DOC) [file pgen.1000252.s004.doc]

**Table S4**

**Nback working memory protective<reference haplotypes**

|  | Voxel-level FDR | T | Z | P  uncorrected | X,Y,Z  mm | K | Region |
| --- | --- | --- | --- | --- | --- | --- | --- |
| Whole brain | 0.038 * | 4.67 | 4.41 | <0.001 | -30, -71, -6 | 10 | Occipital Lobe Lingus BA 18 |
|  | 0.047 * | 4.44 | 4.21 | <0.001 | 45, -49, -18 | 6 | Fusiform Gyrus |
|  | 0.047* | 4.21 | 4.02 | <0.001 | -11, 4, 60 | 15 | Medial Frontal  BA 6 |
|  | 0.047 * | 4.10 | 3.92 | <0.001 | 19, -19, -6 | 2 | Thalamus Ventral Posterior Lateral Nucleus |
|  | 0.047 * | 4.09 | 3.92 | <0.001 | -30, -82, 6 | 12 | Middle Occipital Gyrus  BA 19 |
|  | 0.047 * | 4.01 | 3.84 | <0.001 | 15, 11, 54 | 2 | Superior Frontal Gyrus  BA 6 |
|  | 0.047 * | 3.87 | 3.71 | <0.001 | 26, -86, -6 | 1 | Inferior Occipital Gyrus  BA 18 |
|  | 0.047 * | 3.86 | 3.71 | <0.001 | 34, 0, 48 | 1 | Middle Frontal Gyrus  BA 6 |
|  | 0.047 * | 3.82 | 3.67 | <0.001 | 38, -64, -12 | 1 | Posterior Lobe Cerebellum |
|  | 0.047 * | 3.80 | 3.65 | <0.001 | 38, 52, 12 | 1 | Middle Frontal Gyrus  BA 10 |
|  | 0.047 * | 3.72 | 3.58 | <0.001 | -15, -11, 24 | 1 | Caudate |
|  | 0.047 * | 3.72 | 3.58 | <0.001 | 41, -22, 30 | 1 | Post Central Gyrus |
|  | 0.049 * | 3.70 | 3.56 | <0.001 | -4, 0, 18 | 1 | Caudate |
| ROI caudate  and putamen | 0.053 + | 3.34 | 3.23 | <0.001 | -22, 0, 12 | 36 |  |
|  |  | 2.70 | 2.74 | <0.003 | 22, -4, 12 | 23 |  |

*Threshold set at FDR 0.05 corrected

+Threshold set at 0.05 uncorrected for ROI

Coordinates in MNI space

FDR, false discovery rate; K, cluster; BA, Brodmann area; ROI, region of interest. Coordinates are in MNI space.
