# Supplementary material for: Functional Polymorphisms in PRODH Are Associated with Risk and Protection for Schizophrenia and Fronto-Striatal Structure and Function
Source: PLoS Genet. 2008 Nov 7;4(11):e1000252. doi: 10.1371/journal.pgen.1000252 (PMC2573019; doi:10.1371/journal.pgen.1000252)
Supplement: Table S5 — Demographics. (0.04 MB DOC) [file pgen.1000252.s005.doc]

Table S5

Functional Haplotype Demographics

| **VBM** | reference | risk | protective |
| --- | --- | --- | --- |
| Age* | 30.9, 8.0 | 38.4, 12.4 | 29.7, 7.8 |
| Gender (% male) | 46.9% | 42.9% | 56.3% |
| Wais IQ | 107.0, 8.8 | 110.7, 8.9 | 106.3, 9.6 |

| **Nback** | reference | risk | protective |
| --- | --- | --- | --- |
| Age ** | 31.7, 9.1 | 41.7, 9.9 | 31.7, 9.6 |
| Gender (% male) | 44.4% | 50% | 61.6% |
| Wais IQ | 107.3, 8.7 | 111.1, 9.6 | 106.5, 8.4 |
| Performance | 0.80, 0.16 | 0.74, 0.17 | 0.81, 0.16 |

Mean, s.d.

ANOVA Significantly different at *F=3.687,p=0.027 and **F=3.311, p=0.038

Negative Control Haplotype Demographics

| **VBM** | reference | risk | protective |
| --- | --- | --- | --- |
| Age* | 32.5, 10.1 | 27.0, 7.3 | 33.0, 7.7 |
| Gender (% male) | 56.2% | 45.8% | 40% |
| Wais IQ | 108.1, 9.6 | 107.9, 9.6 | 108.8, 8.0 |

| **Nback** | reference | risk | protective |
| --- | --- | --- | --- |
| Age | 32.5, 11.2 | 27.5, 8.3 | 32.7, 8.3 |
| Gender (% male)** | 61.5% | 33.3% | 37.5% |
| Wais IQ | 108.3, 9.7 | 106.0, 11.1 | 105.9, 8.4 |
| Performace | 0.81, 0.16 | 0.79, 0.16 | 0.80, 0.17 |

Mean, s.d.

ANOVA Significantly different at *F=3.664,p=0.029 and **F=3.781, p=0.026
